# Significance of Natural Scene Statistics in Understanding the Anisotropies of Perceptual Filling-in at the Blind Spot

**DOI:** 10.1038/s41598-017-03713-w

**Published:** 2017-06-15

**Authors:** Rajani Raman, Sandip Sarkar

**Affiliations:** Saha Institute of Nuclear Physics, HBNI, 1/AF, Bidhannagar, Kolkata, 700064 India

## Abstract

Psychophysical experiments reveal our horizontal preference in perceptual filling-in at the blind spot. On the other hand, tolerance in filling-in exhibit vertical preference. What causes this anisotropy in our perception? Building upon the general notion that the functional properties of the early visual system are shaped by the innate specification as well as the statistics of the environment, we reasoned that the anisotropy in filling-in could be understood in terms of anisotropy in orientation distribution inherent in natural scene statistics. We examined this proposition by investigating filling-in of bar stimuli in a Hierarchical Predictive Coding model network. The model network, trained with natural images, exhibited anisotropic filling-in performance at the blind spot, which is similar to the findings of psychophysical experiments. We suggest that the over-representation of horizontal contours in the natural scene contributes to the observed horizontal superiority in filling-in and the broader distribution of vertical contours contributes to the observed vertical superiority of tolerance in filling-in. These results indicate that natural scene statistics plays a significant role in determining the filling-in performance at the blind spot and shaping the associated anisotropies.

## Introduction

When two aligned bars are presented on opposite sides of the blind spot such that the gap fully falls inside the blind spot, the bars are usually perceived as a continuous one. Even though we do not receive any signal related to the bar from the blind spot region, our brain by some means fills the information, which results in a perception of a long continuous bar^1, 2^. This phenomenon is generally referred to as perceptual completion or filling-in. Psychophysical investigations have revealed that the nature of bar filling-in depends on various stimulus attributes (e.g. length, alignment and orientation difference).

In studies related to filling-in at the blind spot^3^, it has been demonstrated that a certain minimum length (extended beyond the blind spot) of bar stimuli is required for filling-in to occur. Moreover, results of investigations show that the minimum length is orientation dependent; and additionally, for the horizontal configuration, relatively shorter length is required for filling-in to occur. Results also revealed that for the identical length, horizontal configuration produced better filling-in over vertical configuration. This phenomenon is referred to as anisotropy in filling-in.

Other related studies^4, 5^ also demonstrated the presence of anisotropy of tolerance in filling-in. However, contrary to the conventional horizontal dominance in filling-in, in this case, vertical dominance was observed; vertical configuration exhibited greater tolerance to the difference in alignment or orientations for perceptual filling-in to occur. This phenomenon is known as anisotropy of tolerance in filling-in. These psychophysical investigations^3–5^ suggest that the perceptual filling-in depends upon stimulus orientation configuration along with the stimulus attributes.

Other than blind spot filling-in, anisotropy has also been observed in other visual phenomena related to orientation perception. Studies with grating stimuli show that visual system is biased toward cardinal (horizontal and vertical) orientation compared to oblique^6^. This effect is known as ‘oblique effect.’ On the other hand, studies involving natural broadband stimuli reveal the opposite where oblique orientations have upper hand over cardinal ones^7–9^. This phenomenon is known as ‘horizontal effect.’ These studies brought out the differences in bias between horizontal and vertical orientation and demonstrated that our visual system favors horizontal configuration over vertical. It has been suggested^6, 8, 10–12^ that the statistics of natural scenes is primarily responsible for the emergence of anisotropy in the orientation perception. Image analysis^8, 10, 13–15^ also supported the fact that the orientation content in natural scenes is biased more towards horizontal over vertical, and the least bias is towards the oblique. This asymmetry raises a logical question whether the orientation-selective neurons in the cortex are influenced by the prevalence of horizontal orientation in the environment. Indeed, it has been demonstrated experimentally^16–19^ that larger cortical area in adult ferret and cat V1 is allocated to processing horizontal information than that allocated to processing vertical information. It implies that comparatively more neurons are devoted to processing information oriented towards horizontal than vertical, i.e., the cortical area contains an over-representation of neurons coding horizontal orientations. Studies using fMRI also show^11, 12^ the anisotropic preference of the human visual cortex to orientation selectivity.

The knowledge of environmental statistics and the related over-representation of neurons in V1 throw some light on the possible causes of anisotropies observed in the perceptual judgments in orientation perception. However, it does not address the phenomenon of perceptual filling-in at the blind spot and the associated anisotropy related to filling-in. In a different context^4^, the role of vernier acuity and elliptical shape of receptive fields of neurons was speculated in the anisotropy of perpetual filling-in. In studies^5^ related to linear and curvilinear filling-in, it was suggested that different processes might be responsible for the anisotropy observed in linear and curvilinear case. Moreover, these studies also speculated different processes for anisotropy observed in different types of tolerances in filling-in. However, these speculations neither explain different processes nor fit with a general computational mechanism of visual processing.

Very recently^20^ it has been shown that bar (shifting and misaligned bar) filling-in phenomena at the blind spot could be explained by considering the inherent prediction correction mechanism of Hierarchical Predictive Coding (HPC)^21^ (as the computational principle of the cortex). It was argued that in the absence of any feedforward information (due to the absence of sensory input corresponding to the blind spot region), top-down prediction dominates the filling-in of the discontinuity. The nature of filling-in, on the other hand, was in accordance with the learned internal model. For proper prediction of the input bar stimuli, it is necessary for the top-down mechanism to predict two separate bars (aligned or misaligned). Instead, in both the cases, top-down mechanism favored the presence of a single continuous bar (resulting in the filling-in at the blind spot), which was the dominant feature of the internal model learned via training with natural images.

These results suggested two very important aspects of filling-in. Firstly, filling-in at the blind spot is the outcome of the prediction-correction mechanism of the cortex, and secondly, the abundance of features present in the natural scene determines the nature of filling-in. These findings are based on investigations involving shifting and misaligned bar stimuli oriented in the horizontal configuration. Therefore, these results cannot address the orientation-specific anisotropies of filling-in at the blind spot, where human observers reported horizontal superiority in filling-in and vertical superiority in the tolerance in filling-in. In a different context, studies with cortical neurons demonstrated its orientation dependent stability in response to selective perturbation induced by adaptation^13^. This behaviour was attributed to the anisotropic distribution of local inputs to the orientation selective neurons, i.e., a narrower distribution of local inputs to the neurons makes it more stable compared to the neurons having a broader distribution of local inputs. It is an important finding that relates the anisotropic distribution of local inputs (to the orientation selective cortical neurons) to its orientation selective stability. However, the significance of these findings concerning anisotropy in the context of filling-in at the blind spot is not clear.

We reasoned that the inherent anisotropy of natural scene could be responsible for the emergence of anisotropy in perceptual filling-in including anisotropy of tolerance in filling-in. We hypothesized that the over-representation of orientation preference in the natural scene contributes to the observed anisotropy of filling-in and the nature of orientation preference distribution determines the observed anisotropy of tolerance in filling-in.

To test these propositions, we have investigated three cases of bar filling-in at the blind spot via simulation studies in a model network^20^ in the light of Hierarchical Predictive Coding scheme. We used expanding, misaligned, and rotating bar as the input stimuli in horizontal and vertical configuration. In response to these input stimuli, the model network exhibited anisotropy of filling-in as well as anisotropy of tolerance in filling-in, which corroborated the findings of psychophysical experiments with human observers.

## Results

**Figure 1:**
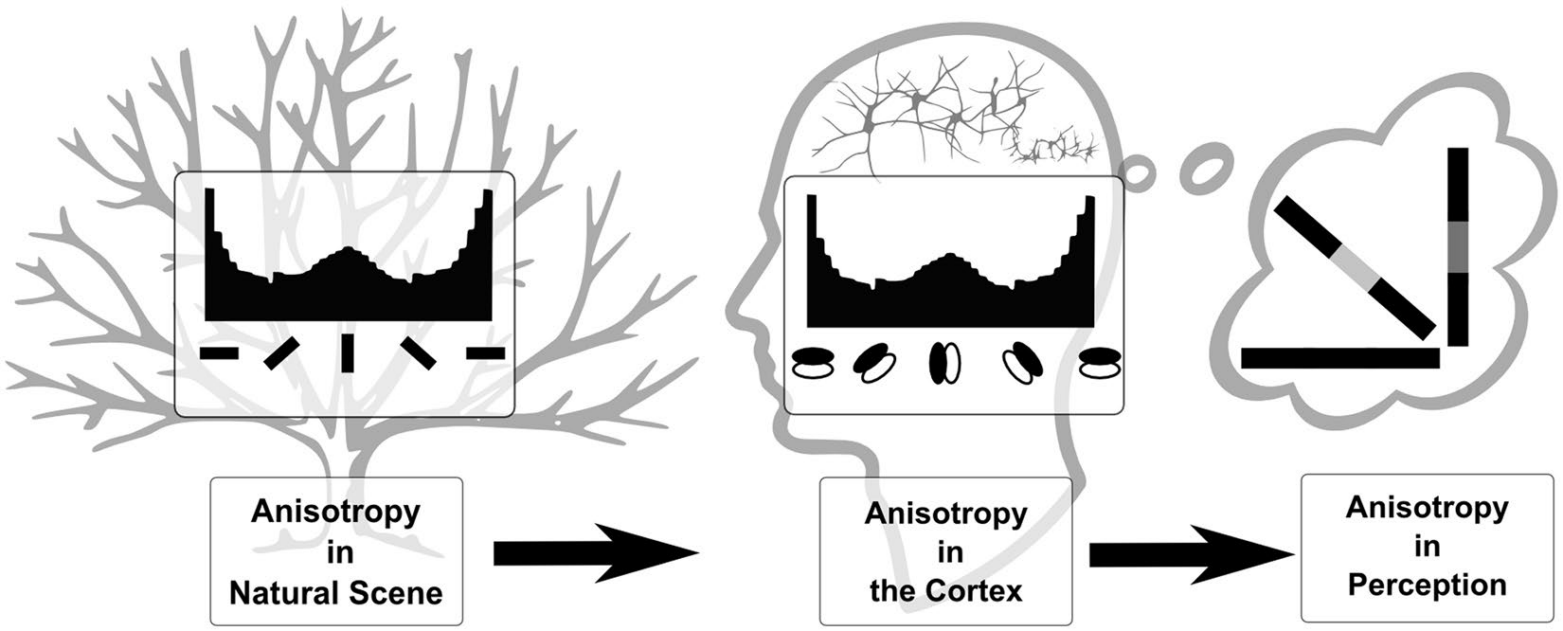
Anisotropy in the natural scene, the cortex, and perception. The aim of this work is schematically presented in this diagram. Here we explored the possible link between the anisotropy in the contours in the natural scene, orientation preference of neurons in the cortex and orientation bias in human perception.

The objective of this study is to test the hypothesis that the prevalence of certain features in natural scenes is capable of providing a mechanistic explanation of anisotropy related to the perceptual filling-in reported by human observers. Our objective is summarised in Fig. 1, where we have schematically depicted the proposition that there is a link between the anisotropy present in the natural scene and the anisotropy reported in perceptual filling-in investigations. This proposition supports the general speculation^6, 8, 10–12^ that orientation anisotropy in natural scene plays a significant role in determining the anisotropy in the cortex as well as the anisotropy in perceptual orientation preference. As a premise, we first explored the capability of the HPC model network to learn (via training) the anisotropic distribution of features present in the natural image. Secondly, we explored whether the learned statistics (learned internal model) could explain the anisotropy in filling-in and the anisotropy of tolerance in filling-in reported in other psychophysical studies.

**Figure 2:**
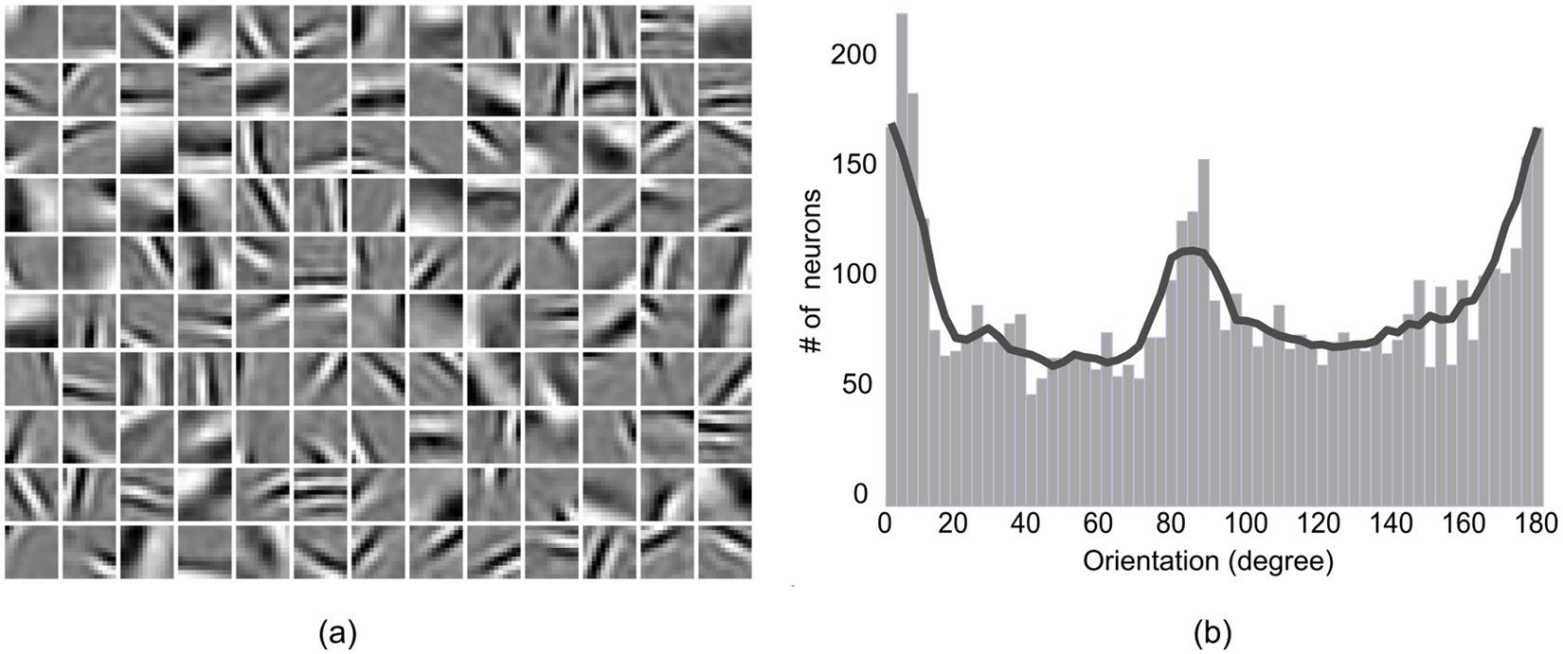
Anisotropy in orientation selectivity. (**a**) Learned weighting profiles of 130 neurons at one of the 9 modules at level 1 after a single training. (**b**) Orientation distribution at level 1 for all the neurons (130 × 9). The envelope (continuous line) is obtained by averaging over 7 bins of the histogram.

The HPC model network considered in this study is similar to the one described in a recent investigation^20^ (details are given in the method section). The network was trained with hundreds of thousands of natural image patches in one cycle. To perform the investigations with statistical rigors, we repeated the training cycle 40 times. As reported in several studies^20–22^, each training set yielded Gabor-like weighting profiles at level 1 (Fig. 2a) (with different orientation and spatial frequency), which resembles the simple cell receptive field at V1. Level 2 weighting profiles resemble more abstract features (corner, curves, long bar, and so on) as reported in recent studies^20^.

To investigate the presence of any anisotropy, we measured the orientation tuning distribution of the trained neurons in V1. To do these, we utilized bar stimulus of different orientation and frequency and determined the orientation tuning of a particular neuron by registering their optimal response. Figure 2b shows the distribution of orientation tuning of neurons in V1. It is evident from the distribution that larger number of neurons are oriented towards the horizontal direction, followed by vertical and then non-cardinal orientation. This anisotropic distribution is very much in-line with the reported anisotropy of orientation distribution in natural scenes^10, 13, 23^ and orientation tuning distribution of neurons in primary visual cortex^11, 12, 17, 19, 24^.

### Anisotropy in filling-in

**Figure 3:**
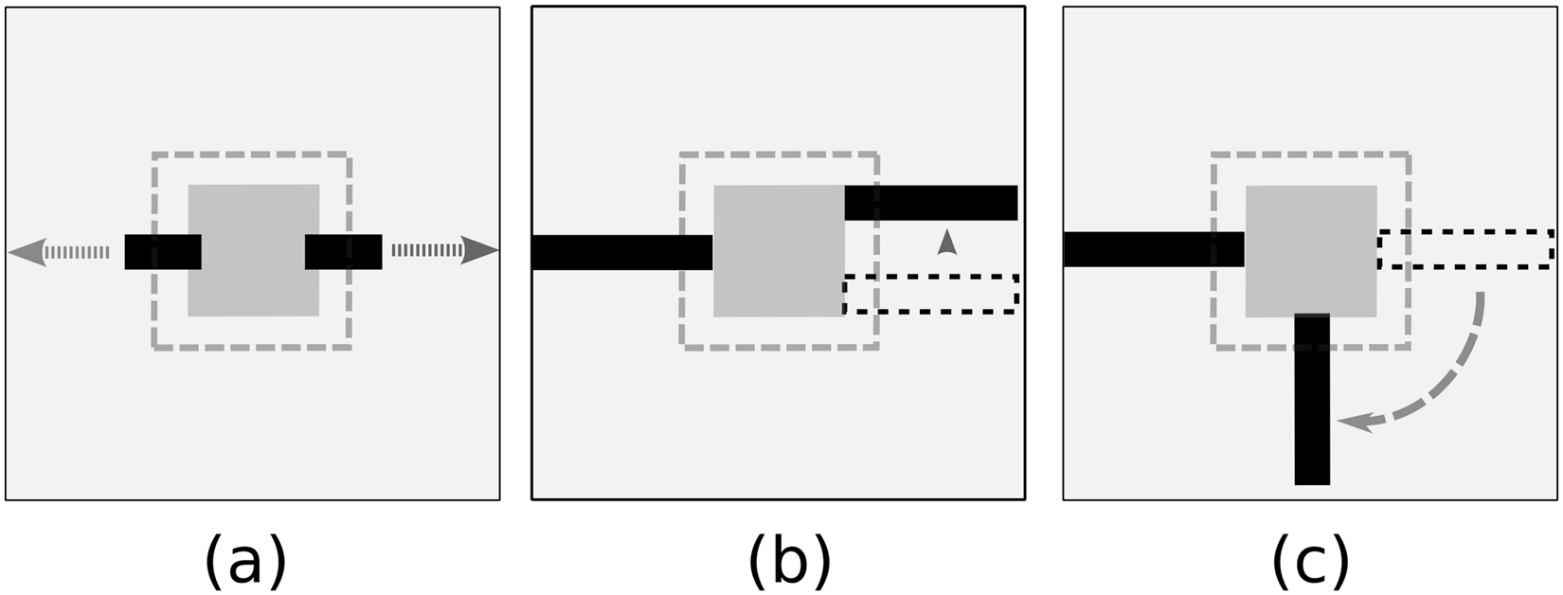
Stimuli. (**a**) Expanding bar stimulus: Two bar stimuli are shown at the opposite end of the blind spot shown as a gray square (8 × 8 pixels) in the center. The dotted square (12 × 12 pixels) denotes the area exposed to the central module (called BS module) of one of the nine level 1 modules (see Methods). One end of both bars is fixed inside the blind spot, whereas other ends are free to expand in sync in steps of one pixel in opposite directions. Bar extension is measured from the border of the blind spot. (**b**) Misaligned bar stimulus: The bar at the left side of the blind spot is fixed while the bar on the right side is free to move in the vertical direction in steps of one pixel every time. (**c**) Rotating bar stimulus: In this case, the bar on the left side is fixed but the bar at the right side can rotate in steps of 10 degrees.

**Figure 4:**
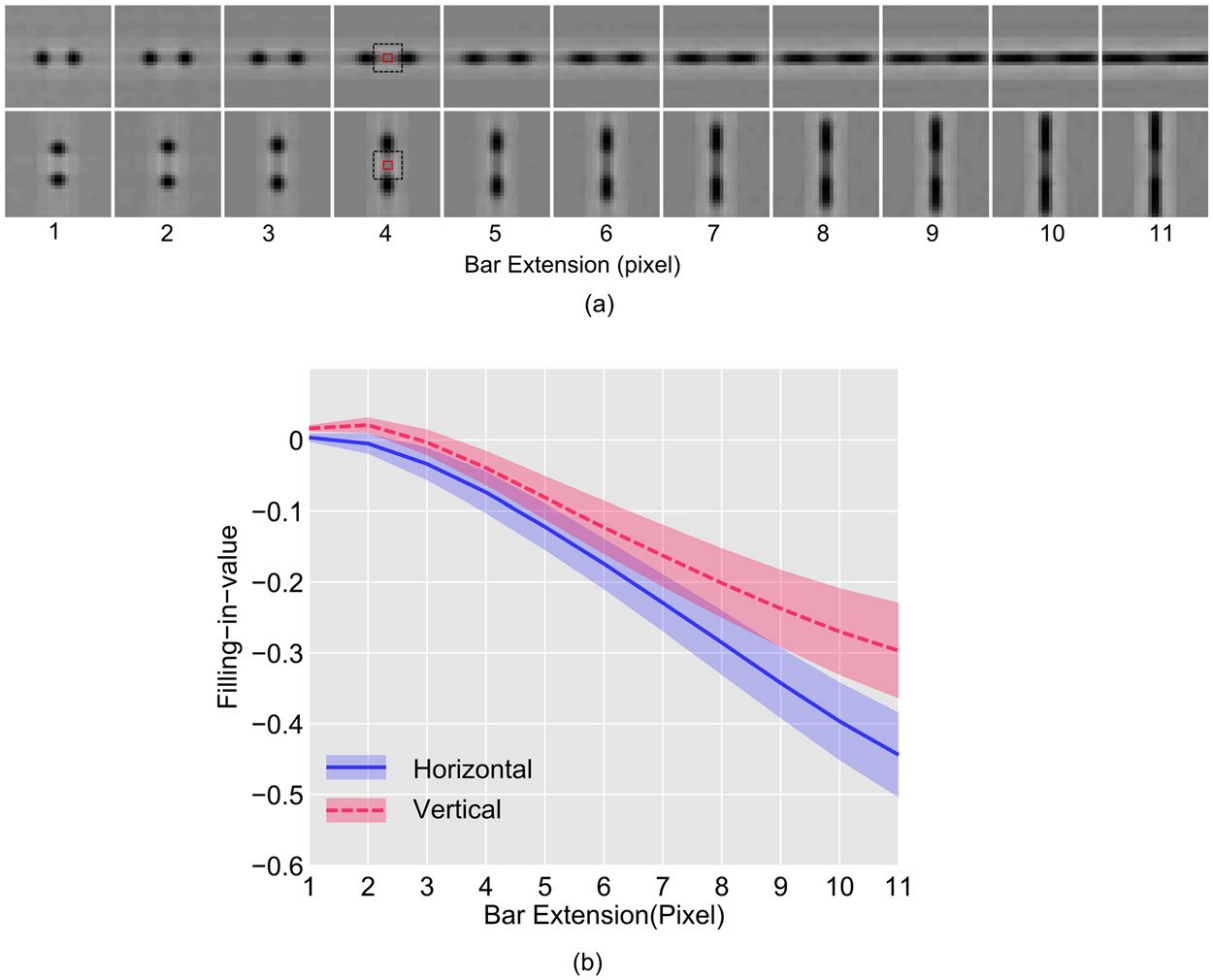
Anisotropy in filling-in. (**a**) Perceptually equivalent images are shown, which are generated from the response of PE neurons while the network was stimulated with stimuli depicted in Fig. 3a. The dotted black square (in the fourth column) indicates the position of the blind spot and the red square indicates the area (2 × 2 pixel) from where the average filling-in-value is obtained. The same convention is followed for all the images. (**b**) The plots of ‘filling-in-value’ in BS area of the images in (**a**) are ﻿shown﻿ as a function of bar extension measured from the edge of the blind spot. The lines represent the average, and the shaded portion indicates the standard deviation for the 40 training set.

To investigate the anisotropy in filling-in, the learned network was exposed to a pair of expanding bar segments, placed as shown in Fig. 3a, oriented in the horizontal direction. One end of both bars was fixed and other ends were free to expand together in sync as described in the Fig. 3a. The network was also stimulated with stimuli oriented in the vertical direction (not shown). The responses of PE neurons were recorded as a function of bar extension (length) for both orientation configurations. This process was repeated 40 times with 40 different training cycles. Investigations with different training can be considered analogous to the psychophysical investigation performed on different participants (human), which leads to more statistical rigors in results. All the subsequent investigations reported in this study follow the same number of repetitions. From these simulated responses, equivalent “perceptual images” were reconstructed, which are shown in Fig. 4a for both horizontal (top row) and vertical configurations (bottom row).

To quantify the filling-in, grayscale values in the middle (central 2 × 2 pixel wide region inside the blind spot, indicated by the small red square in Fig. 4a) of the perceptual image were averaged. We defined this average as the ‘filling-in-value’, where more negative ‘filling-in-value’ indicates better filling in. We obtained this response values from all the perceptual images corresponding to 40 training for the given attributes (bar extension and configuration).

Figure 4b shows the plot of ‘filling-in-value’ as a function of the bar extension for both configurations (horizontal and vertical). Inspection of Fig. 4b shows that the filling-in starts improving rapidly (more negative) when the length of the expanding bar exceeds a certain minimum. It can be visualized from the perceptual images (Fig. 4a) where beyond a certain minimum length, the bars appear continuous. This result exhibits the ‘minimum-length requirement’^3^ property of filling-in. The comparative plots of filling-in-value for horizontal and vertical configuration in Fig. 4b show that for a particular filling-in-value the minimum critical length needed for the onset of filling-in would be lesser for the horizontal configuration. In other words, for the equal bar extension, the filling-in performance is better (more negative ‘filling-in-value’) for the horizontal case. This anisotropic property is in agreement with the results of psychophysical studies^3^.

To validate our results, a two-way ANOVA was conducted that examined the significance of the effect of bar extension and the configuration (horizontal and vertical) on the filling-in-values. We found that the effect of extension [F (10,858) = 933.93, p = 0)], configuration [F (1,858) = 585, p = 0)], and, the interaction between them [F (10,858) = 24.09, p = 0)] was significant. Moreover, a post hoc Tukey test showed that the horizontal and vertical configuration differed significantly at p = 0.

### Anisotropy of tolerance in filling-in

#### Anisotropy of tolerance in filling-in for misaligned bar

**Figure 5:**
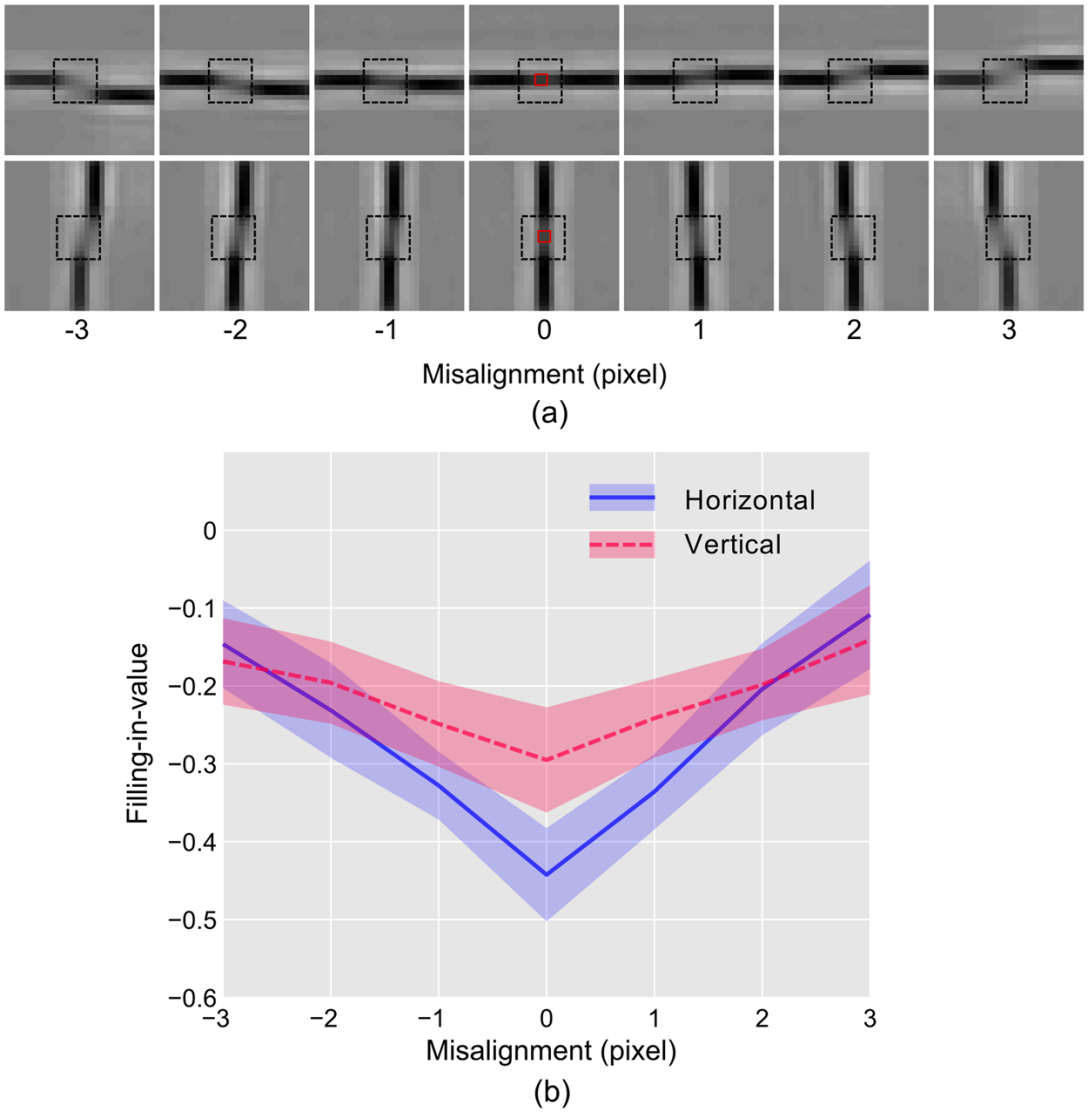
Anisotropy of tolerance in filing-in for misaligned bar. (**a**) Perceptually equivalent images are shown, which are generated from the response of PE neurons while the network was stimulated with stimuli depicted in Fig. 3b. (**b**) The plots of ‘filling-in-value’ in BS area of the images in (**a**) as a function of misalignment between the bars are shown. Convention for lines and the shades are as described in Fig. 4b.

For this study, the model network was exposed to a pair of bar segments placed on both sides of the blind spot, and this is repeated separately for horizontal and vertical configuration. The arrangement for the horizontal case is shown in Fig. 3b. One bar was kept fixed at one side of the blind spot while the position of the other one was shifted vertically in small steps to vary the misalignments. The response of PE neurons in BS module was recorded with changing misalignment, and the perceptually equivalent images were generated from these responses, which are shown in Fig. 5a (top row). Likewise, the images generated for the vertical configuration are shown in Fig. 5a (bottom row).

The images show that, in both configurations, the filling-in is best in the case of perfect alignment but deteriorates with increasing misalignment. Inspection of ‘filling-in-value’ plotted in Fig. 5b show that it is more negative (better filling-in) for the horizontal configuration compared to that of the vertical one, which is the signature of anisotropy in filling-in, as we have already discussed in the previous section. Moreover, we can also observe that the slope of the curve is higher for the horizontal case. It indicates that the rate of change of the ‘filling-in-value,’ for the horizontal orientation, is more sensitive to the change in misalignment. In other words, filling-in, in the case of vertical orientation, is more tolerant to misalignment compared to that of the horizontal orientation. This behaviour could be considered as a signature of anisotropy of tolerance in filling-in.

A two-way ANOVA was conducted that examined the significance of the effect of bar misalignment and the configuration on the filling-in-values. We found that the effect of misalignment [F (6,546) = 175.91, p < 0.001)], configuration [F (1,546) = 81.96, p < 0.001)], and, the interaction between them [F (6,546) = 26.53, p < 0.001)] was significant. Additionally, a post hoc Tukey test showed that the horizontal and vertical configuration differed significantly at p = 0.

#### Anisotropy of tolerance in filling-in for disoriented bar

**Figure 6:**
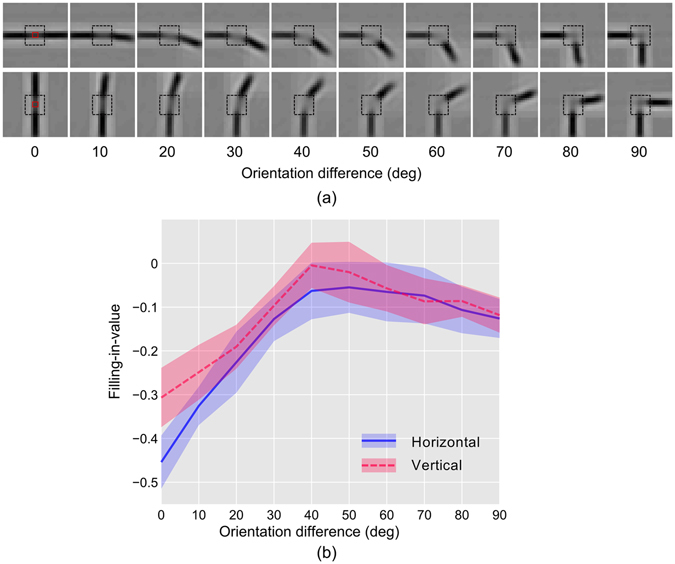
Anisotropy of tolerance in filing-in for disoriented bar. (**a**) Perceptually equivalent images are shown, which are generated from the response of PE neurons while the network was stimulated with stimuli depicted in Fig. 3c. (**b**) The plots of ‘filling-in-value’ in BS area of the images in (**a**) as a function of orientation difference between the bars are shown. Convention for lines and the shades are as described in Fig. 4b.

The focus of the study was to investigate the anisotropy of tolerance in filling-in for orientation difference of two bar segments placed on both sides of the blind spot in horizontal and vertical configuration. The configuration for the horizontal case is shown in Fig. 3c. The stimulus consisted of a fixed bar and a rotating bar. The fixed bar was placed horizontally for the horizontal configuration and vertically for the vertical configuration. The other bar, the test bar, was rotated in steps of 10 degrees from the aligned position (0 degree difference in orientation) to the perpendicular position (90 degree difference in orientation). The perceptual images, generated from the recordings of PE neurons, are shown in Fig. 6a for both horizontal (top row) and vertical cases (bottom row).

As expected, the filling-in performance is better for the aligned bars, but it deteriorated with increasing difference in orientation (Fig. 6b) in both configurations. It is also evident that the ‘filling-in-value’ is more negative (indicating better filling-in), in horizontal case, throughout the entire range of difference (in orientation) from 0 degrees to 60 degrees and after that, the difference becomes indistinguishable. The results show that the horizontal configuration favors filling-in but exhibit more sensitivity to the changes in orientation difference (less tolerant); on the other hand, the vertical configuration is little less favorable for filling-in but is less sensitive to the changes in orientation difference (more tolerant).

A two-way ANOVA was conducted that examined the significance of bar disorientation and the configuration on the filling-in-value. We found that the effect of disorientation [F (9,780) = 334.4, p < 0.001)], configuration [F (1,780) = 104.66, p < 0.001)], and, the interaction between them [F (9,780) = 13.12, p < 0.001)] was significant. Additionally, a post hoc Tukey test showed that the horizontal and vertical configuration differed significantly at p = 0.

#### Comparison with the psychophysical results

**Figure 7:**
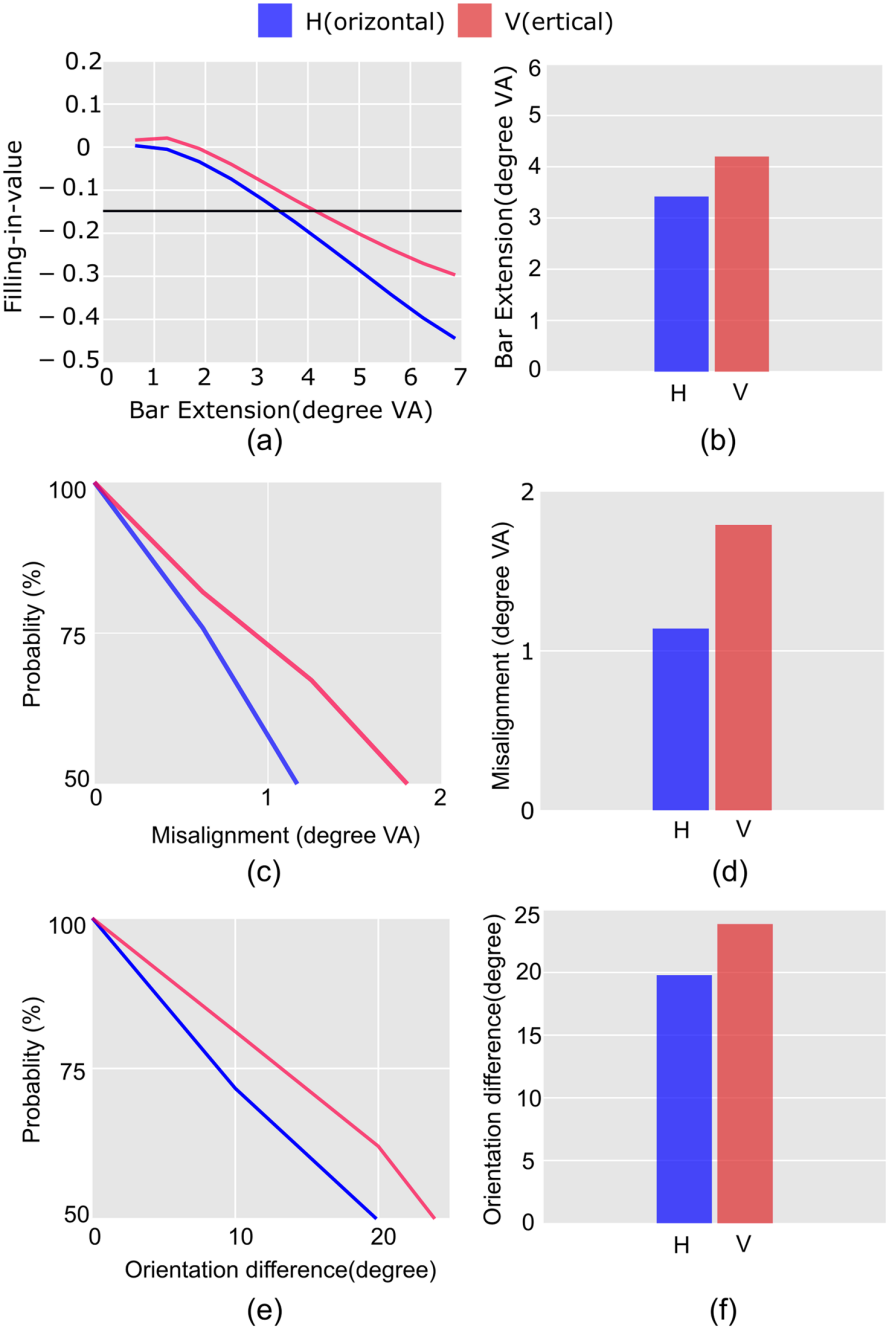
Comparison with psychophysical results. Results of Figs 4b, 5b and 6b are redrawn in (**a**), (**c**) and (**e**) respectively for the comparison. The visual angle is represented as VA in the plots. (**a**) The horizontal line represents the threshold corresponding to the 50% of the maximum filling-in-value for vertical configuration, and the estimated bar lengths corresponding to this threshold are plotted as bars in (**b**). (**c**) Normalized plots, as explained in the text, for the positive misalignments are presented, which continued from the 100% to 50% probability (artificial threshold). The amount of misalignment at this threshold for the horizontal and vertical cases are shown as bars in (**d**). (**e**) Similar normalized plots for orientation difference are shown here and estimated orientation difference at 50% threshold is shown as bar plots in (**f**).

For the purpose of direct comparison with psychophysical results^4, 5^, we have redrawn our results (Figs 4b, 5b and 6b) in Fig. 7 taking into account the concept of visual angle (VA) and a threshold. In our study, the extent of the model blind spot is 8 × 8 pixels. On the contrary, if we approximate the blind spot to be a square region, the average size of the spot is 5 × 5 degree^25, 26^. Therefore, we have used a scaling factor of 0.625 for converting pixels to degrees. Additionally, we have introduced an artificial threshold (at 50%), which is used to obtain quantitative estimates. Bar diagrams compatible for comparison with psychophysical experiments are plotted on the right of each of the plots.

In psychophysical investigations, the anisotropy in filling-in (discussed in Fig. 4) was measured using staircase method^3^. On the contrary, in our study, we have measured the filling-in-value which directly corresponds to the activity of the PE neurons of our model network. Change in the activity of these neurons, for exposure to different stimuli, encodes properties of the filling-in process. As shown in Fig. 4b, in our model network, neurons exhibited higher activity (more negative filling-in-value) when exposed to horizontal bar stimulus compared to the activity induced by vertical bar stimulus for a given bar length. Therefore, bar length that induces similar levels of neural activity will be different for different configuration (horizontal or vertical), and this may provide an estimate of anisotropy. We have estimated these lengths (‘minimum length requirement’) by considering a threshold at 50% filling-in-value corresponding to the vertical case (in red) as shown in Fig. 7a. The estimated bar lengths are plotted as bars in Fig. 7b that are similar to the results reported in experiments^3^. We did not consider a threshold corresponding to the 50% filling-in value in the horizontal case because that would have estimated longer bars, whereas our focus is to find the minimum lengths of bars.

For the presentation of results related to the tolerance in filling-in, we have conceived a general notion of the tolerance as a rate of change of filling-in-value, with increasing difference in attributes. Faster change (higher rate) will indicate lesser tolerance, and this is advantageous because one can predict the tolerance by inspecting the slope of the curve representing the changing filling-in-value, which is directly available from the simulation study. Several psychophysical studies^4, 5^, on the other hand, have defined tolerance in filling-in as the maximum difference in attribute above which filling-in is not perceivable with certainty. This definition is more compatible with the outcome of psychophysical experiments. Therefore, a different presentation of our results is necessary for direct comparison with psychophysical findings.

For comparison, we have normalized the results shown earlier in Figs 5b and 6b. The normalization is performed by dividing the filling-in values represented by a given curve (for a specific configuration) by the magnitude of the maximum filling-in value for that specific case, and this is repeated for each of the plots separately. To compare with the psychometric plot of psychophysical results we have, in the resultant plots, considered −1 to represent 100% probability and 0 to represent 0% probability. Additionally, following the general convention where the y-axis ranges from minimum probability (at the bottom) to the maximum (at the top), we have inverted our plots which are shown in Fig. 7c and e. These normalized plots range from 50% probability at the bottom to the 100% probability at the top and tolerances are estimated from these plots. We have introduced an artificial threshold at the 50% probability, where it is assumed that beyond this threshold filling-in cannot be perceived with certainty.

In line with the definition of tolerance (maximum difference for which filling-in cannot be perceived with certainty) compatible with psychophysical experiments, tolerances are represented as vertical bars in Fig. 7d and f. Estimated lengths for misalignment shown in Fig. 7d corroborate the experimental findings presented in the psychophysical study^4^ for the vertical as well as for horizontal configurations. The estimated orientation difference for horizontal and vertical configurations are shown in Fig. 7f. In this plot, the tolerances are ~19 degrees and ~24 degrees for horizontal and vertical configuration respectively while the psychophysical results^4^ provides these value as ~40 degrees and ~55 degrees. Though the magnitude of tolerances obtained in our investigation differs from the tolerances reported in psychophysical experiments, it is interesting to observe that the ratio (1.26) of vertical to horizontal tolerance is very close to the value (1.37) obtained experimentally. It shows that qualitatively our results agree well with the experimental findings. These results presented in Fig. 7d and f clearly show the vertical dominance in the case of tolerance of filling-in for misalignment and as well as for disorientation.

Filling-in phenomenon for misaligned bars was reinvestigated recently^5^ in the context of linear and curvilinear completions at the blind spot. In this study, perceptual completion was defined as the case where participants perceived continuous bar irrespective of the apparent shape being straight or curved. This definition is identical to the misalignment case we have considered in this article as well as the one reported earlier^4^. Though the tolerance of misalignment reported in later study^5^ is higher approximately by a factor of 2 than the results reported eariler^4^, the vertical dominance in filling-in is preserved and the ratio of vertical to horizontal misalignment remained very close to that reported in psychophysical study^4^. Therefore, our results related to misalignment investigation to some extent corroborates the results reported in psychophysical study^5^.

The common asymptotic shape, as observed in psychometric plots, near 100% probability^5^, is not apparent in our plots. The most plausible reason is the lower resolution we have achieved in our simulation, where we have considered a 8 × 8 pixels wide blind spot that provided 4 data points corresponding to four misalignments. For the same limited resolution, the results of collinear experiments^5^ could not be discussed with the results of our investigation. The collinear filling-in has been shown for the very narrow misalignment which is not possible to investigate in the current context. However, a model network with a better resolution could be able to shed some light on these phenomena.

#### Relation between natural image statistics and filling-in at the blind spot

How anisotropy, then, arises from the response of the model network? We have shown (Fig. 2b) that, in agreement with natural scene statistics, the distribution of the orientation preference of the learned receptive fields at V1 reflects the over-representation of neurons tuned towards horizontal orientation. This result demonstrates that the model network could encode the anisotropies of natural scene statistics through learning. In a separate study^20^, it has been suggested that the likelihood of filling-in of features (bars with different attributes) is guided by its likelihood of occurrence in the natural scene. Features that are more frequent tend to be more likely candidates for filling-in. In this perspective, we argue that the over-representation encoded by the learned receptive fields at V1 dominates the prediction at the blind spot that leads to filling-in of discontinuity. This is plausible because in the absence of the feed-forward connections (in the network representing blind spot region) top-down predictions biased by the learned internal model dominates. Thus, the prevalence of horizontally oriented features (lines, bar and the like) in the learned internal model results in the superiority of horizontal features in filling-in. This is reflected as more negative ‘filling-in-value’ in all three horizontal cases (blue line) in Figs 4b, 5b and 6b.

How vertical superiority of tolerance in filling-in arises? The nature of variation in filling-in-value, shown in Fig. 5b (or Fig. 7c) and Fig. 6b (or Fig. 7e), can be explained by taking into account the orientation tuning distribution of neurons shown in Fig. 2b. Inspection of Fig. 2b reveals that neurons tuned toward horizontal orientation have a higher population and sharper distribution. In comparison, neurons tuned toward vertical orientation have a relatively lower population and relatively broader distribution. The sharper distribution (and higher population) of neurons tuned toward horizontal orientation results in a more specific estimate for filling-in that would be less tolerant despite the fact that better filling-in will be observed for that orientation. On the other hand, broader distribution (and lower population) of neurons tuned toward vertical orientation results in higher tolerance and the lesser response results from the comparatively lower population. Therefore, in the case of horizontally oriented stimuli, the filling-in performance deteriorates at a faster rate with increasing difference in stimulus attributes compared to that of vertically oriented one.

These arguments can be readily put forward for explaining the anisotropy of tolerance in filling-in for disoriented bar stimuli (Fig. 6). For a given configuration (horizontal or vertical), the rotating segment of the stimuli makes varying angles with the fixed segment. Because of this, the filled-in section that resides inside the blind-spot will have to be aligned at varying angles either toward vertical or horizontal depending on the configuration. For every angle (0 to 90°), neurons having the similar orientation preference matching that of the filled-in section (in the blind spot) that connects the pair of bars will be activated for filling-in. For horizontal configuration, neurons having horizontal orientation preference as well as neurons having close to horizontal orientation preference are activated (depending on the stimuli in Fig. 6b). Because of the sharper distribution of neurons with orientation preference toward horizontal, a smaller orientation difference (with the horizontal) of the rotating bar will activate a certain population of neurons with similar orientation sensitivity. However, this population will be much smaller compared to the population that has been deactivated due to the increase in orientation difference. The deactivation will result in a larger decrease in response of the neurons, which is reflected as a faster decrease (lesser tolerance) in responses with increasing stimulus deviation from the horizontal orientation. Similar arguments can be given to explain the slower decrease (greater tolerance) in responses of neurons (because of broader distribution) in the case of vertical configuration.

In the case of misaligned bar investigation (Fig. 5), one bar is kept fixed, and the other is shifted (either vertically or horizontally) to simulate the varying amount of misalignment. Because of this, the filled-in section of the pair of bars (inside the blind-spot) will have to be aligned at varying angles either toward vertical or horizontal depending on the configuration. For every misalignment, neurons having orientation preference similar to that of the filled-in section become activated for filling-in. Therefore, as discussed before, the filling-in-value will be determined by the population of neurons tuned to a specific orientation and the nature of variation (with increasing misalignment) will be determined by the width of the distribution of neurons. This is reflected as better filling-in (more negative filling-in-value) and faster deterioration in filling-in with increasing difference in attributes in case of the horizontal configuration shown in Fig. 5b.

From the preceding discussions, it is evident that the predominance of horizontal contours in natural scene results in better filling-in operation in all three cases considered, and this is reflected as more negative filling-in-value as shown in Figs 4, 5 and 6 (in blue). On the other hand, broader distribution of vertical contours results in a more tolerant response in filling-in operation with increasing difference in attributes, which is reflected in the curves (in red) with shallower gradient depicting the changing filling-in-value in Figs 5 and 6.

**Figure 8:**
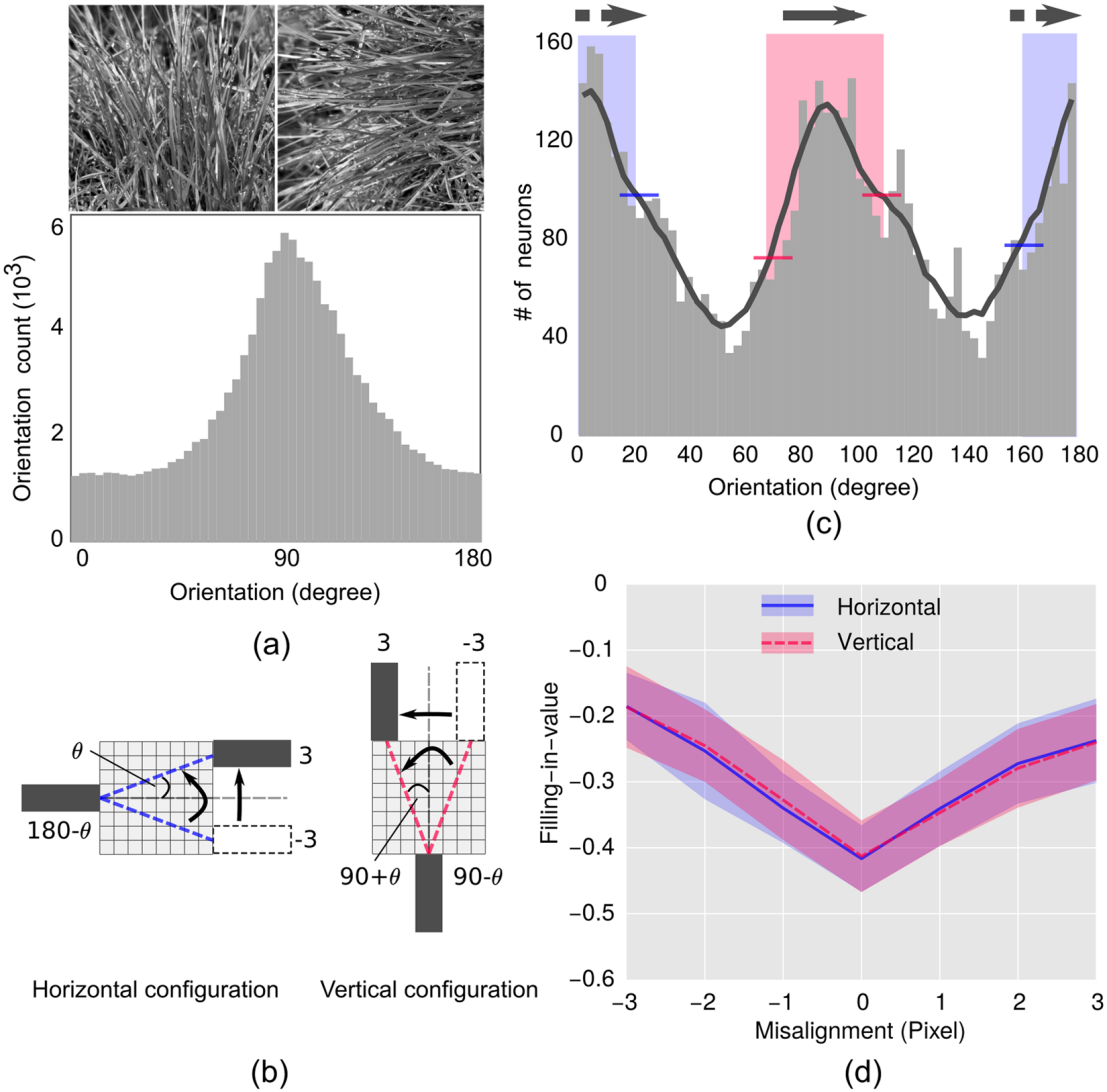
Validation Investigation. (**a**) Natural images with asymmetric orientation distribution are shown. The upper-left image mainly possesses contours with a bias towards vertical orientation. The histogram exhibiting this property is shown below. The upper-right image is 90 degrees rotated version of the left one (histogram is not shown). (**b**) A detailed schematic of the misaligned bar study conducted in horizontal and vertical configuration is presented. The moving bar was shifted by a maximum amount of 3 pixels on both sides of the mean (aligned) position. For the horizontal configuration it moved upward from the bottom, and for the vertical case, it moved leftwards. The angular deviation of the filled-in portion (represented by dotted line inside the BS) can be evaluated from $\theta$ = tan^−1^(position of the moving bar in pixels/8). (**c**) Orientation distribution of trained neurons at level 1 is plotted. The continuous line (black) plot is the envelope of the histogram, which was obtained by convoluting the histogram, averaging over 7 bins. The shaded regions around horizontal (in blue) and vertical (in red) orientation indicate the population of neurons that is likely to be activated for filling-in when the moving bar is displaced by an amount $\pm 20$ degree ($\theta$ = tan^−1^(3/8) ~ 20 degrees) around the mean position. The difference in height between red lines (blue lines) across this smoothed plot is to indicate the neuronal density difference for the maximum misalignment (20 degrees) around the vertical orientation (horizontal orientation). The arrows above the shadowed regions indicate the direction of the moving bar. (**d**) Plots of ‘filling-in-value’ as a function of misalignment obtained from the response of the network are drawn. Convention for lines and the shades are as described in Fig. 4b.

Does the model HPC network predict filling-in-value in accordance with the statistics of natural images it was trained with? To validate these conclusions, we have repeated investigations with misaligned bar stimuli (Fig. 5) with a natural image having vertical orientation superiority with asymmetric distribution of contours and its 90° rotated version, which is shown in Fig. 8a. The distribution of orientation content of the upper-left image is shown at the bottom of Fig. 8a. We have evaluated the orientation at each pixel (upper left image in Fig. 8a) from the direction of the local gradient (of the grayscale image), which was evaluated from the arc tangent of partial derivative (in 3 × 3 kernel) in the vertical direction divided by the value in the horizontal direction.

The distribution reveals the dominance of vertical contours and an asymmetric distribution around the dominant orientation (90 degrees) with a sharper rise (left side) and a slower fall (right side). Training with these two images produced an orientation preference of V1 neurons as shown in Fig. 8c, where the neurons are equally sensitive to cardinal orientations and possessed similar distributions around cardinal directions, which nearly preserved the asymmetries of the original image (Fig. 8a). This similarity resulted in an equal filling-in response as shown by the superimposed curves (representing filling-in-values) in Fig. 8d. Despite the fact that the distributions are similar, close inspection of Fig. 8c reveals that the distributions, centered around cardinal angles, are asymmetric exhibiting a sharper rise at the left side and a comparatively slower fall at the right side. This asymmetry implies that as long as the moving bar is aligned at 180 − *θ* (90 − *θ*) (Fig. 8b), the filling-in-value altered at a faster rate with the angle and when it was aligned at 180 + *θ* (90 + *θ*), the filling-in-value altered at a comparatively slower rate. This is visible in the plot shown in Fig. 8d as faster rise on the left and a slower rise on the right side. From these results we conclude that the filling-in-value predicted by the model HPC network is in accordance with the statistics of images used for training, where the absence of anisotropy in the contours tuned to cardinal orientations results in equal filling-in response; and similar distribution of cardinal orientations results in similar gradient in the changing filling-in-value with increasing difference in the attributes.

## Discussions

Our study suggests that natural scene statistics plays a significant role in determining the anisotropy in perceptual filling-in including the anisotropy of tolerance in perceptual filling-in at the blind spot. Over-representation of horizontal contours in natural scene biases the orientation preference of neurons in V1, and that is possibly responsible for the emergence of anisotropy, which is reflected as a horizontal preference in perceptual filling-in operation. The width of the distribution of orientation preference, on the other hand, determines the anisotropy of tolerance in filling-in, where the broader distribution of vertical contours in natural scene possibly contributes to the greater stability towards vertical orientation in perceptual filling-in operation.

These results demonstrate that there is a link between the orientation anisotropy in the contours in the natural environment, orientation preference of neurons in V1 and orientation bias in the perceptual filling-in at the blind spot. Our result supports the general speculation^6, 8, 10–12^ that orientation anisotropy in natural scene plays a significant role in determining the anisotropy in the cortex as well as the anisotropy in perceptual orientation preference.

Firstly, we show that the model HPC network, which mimics the prediction-correction computational paradigm of the cortex, is capable of building an internal model of the outside environment by learning the statistics of natural scenes it is exposed to. This is reflected by the fact that the orientation preference, as well as the distribution of orientation preference of model neurons in V1, is very similar to the predominance of horizontal contours and their distribution in the natural environment. The plausibility of this paradigm can be established with the help of several previous findings. A recent survey^16^, in the physiological domain, involving cells in the cat’s striate cortex indicates the preferential bias of cells towards horizontal orientation. Imaging studies also revealed^17–19^ the preference of higher percentage of the area of the exposed visual cortex towards horizontal orientation compared to vertical. Innate specification along with prolonged exposure to an anisotropic environment during development is believed to be responsible for the emergence of over-representation of horizontal orientation preference of these neurons. In the psychophysical domain, correspondence between the horizontal bias in human visual processing and the anisotropy in the natural scene has been reported^8, 9^. A detailed survey in this work also shows the prevalence of horizontal contours in a typical natural scene compared to vertical contours. In a recent study, it has been demonstrated that visual orientation perception reflects the knowledge of environmental statistics^6^. In this work, the estimated internal model of human observers was found to match the orientation distribution measured in photographs of environment though the difference between horizontal and vertical was not addressed.

Secondly, our investigations reveal that the anisotropy in orientation preference (horizontal) of V1 neurons results in the similar anisotropy in the filling-in performance and the distribution (sharper or broader) of cardinal neurons results in the anisotropy of tolerance in filling-in performance. What is the biological plausibility of such a scheme? In an imaging study^13^, it has been shown that in V1 the distribution of inputs to the cardinal neurons is narrower compared to those of oblique neurons. When exposed to selective perturbation induced by adaptation (oriented away from the neuron’s preferred orientation), cardinal neurons exhibited greater stability compared to the neurons tuned to oblique orientation. This is attributed to the fact that because of the narrower distribution of local inputs to the cardinal neurons, an adaptive stimulus would stimulate a fewer number of neurons in the vicinity compared to that of the neurons tuned to oblique orientation. This demonstrates that the width of the distribution (of neurons) plays a significant role in determining the responses when stimulated away from the preferred orientation. From a different perspective it indicates that for neurons having narrower distribution, a much greater change in response will be observed with increasing deviation of the stimulus orientation from the neuron’s preferred orientation. This implies greater sensitivity and therefore, lesser stability in the present context. Comparatively, neurons having broader distribution will be less sensitive (more stable). This is similar to the findings of our observation. Evidence in favour of larger neural population preferring horizontal orientation (compared to vertical) have also been found in several physiological studies^16–18^, as discussed earlier.

In studies on filling-in completions at the blind spot^4^, it was speculated that there might be different anisotropic process responsible for different kinds of anisotropy observed in different (misalignment, disorientation, and luminance difference) filling-in investigations e.g., it was speculated that the anisotropy in misalignment experiment might have arisen from the anisotropy in vernier acuity. Alternatively, studies^5^ related to linear and curvilinear filling-in for misaligned bar suggest the possible involvement of different processes for the observed anisotropy. Here in this study, we have proposed a possible alternative explanation in terms of a unified principle based on the role of natural image statistics. We have demonstrated this in filling-in investigations involving misaligned and disoriented bar stimuli. Results of our studies also suggest that the anisotropy in vernier acuity might have its origin in the statistics of natural scenes. Evidence in support of these suggestions can be found in a related work^27^, where it was argued that the vernier misalignment could be discussed on the premise that the average orientation of a misaligned pair of abutting lines differs from that of the aligned lines. Vernier acuity preferring horizontal directions over the vertical including the cardinal over the oblique has been demonstrated in this work.

We speculate that the horizontal superiority^4^ in the tolerance of luminance difference could be discussed in terms of statistics of the natural scene. Luminance is a surface property, and, therefore, for proper inference, the cortex should be capable of encoding 3D surface information efficiently. In a recent study^28^, it has been shown that disparity neurons are capable of encoding statistics of the natural scene. Studies^29^ also show that the pair-wise functional connectivity between the disparity tuned neurons in V1 matches the anisotropic distribution of correlation between disparity signals in the natural scene. Though, these studies mainly concentrated on the cardinal vs non-cardinal aspect of the anisotropy, a close inspection of the plots indicate a broader distribution of the horizontal features. This broader distribution in disparity signal (or pair-wise connectivity) could be linked to the horizontal superiority in the tolerance of luminance difference. Some supportive evidence can be found in a recent work^30^ showing that relative luminance and binocular disparity preferences are correlated in accordance with the trends of natural scene statistics. These studies suggest a possible link between the anisotropy in the disparity signal and the relative luminance. In future work, incorporation of surface representation in the internal model in the HPC framework might explain the anisotropy in luminance difference.

In this work, we have studied the origin of anisotropy in perceptual filling-in in a simple standard linear Hierarchical Predictive Coding network. Because of this, our findings could only explain the possible reasons responsible for the emergence of anisotropy in filling-in reported by human participants, but a quantitative comparison with psychophysical results is not straightforward. In the present context, however, what matters is that given the statistical information of the input stimuli derived from natural images, the network was able to predict the anisotropy in perceptual filling-in at the blind spot. The findings, in this work, offer new insights into the role of natural scene statistics and suggest what is possibly the first systematic bridge linking anisotropy in three levels: natural environment, visual cortex, and perceptual filling-in at the blind spot.

## Methods

### Standard hierarchical predictive coding (HPC)

**Figure 9:**
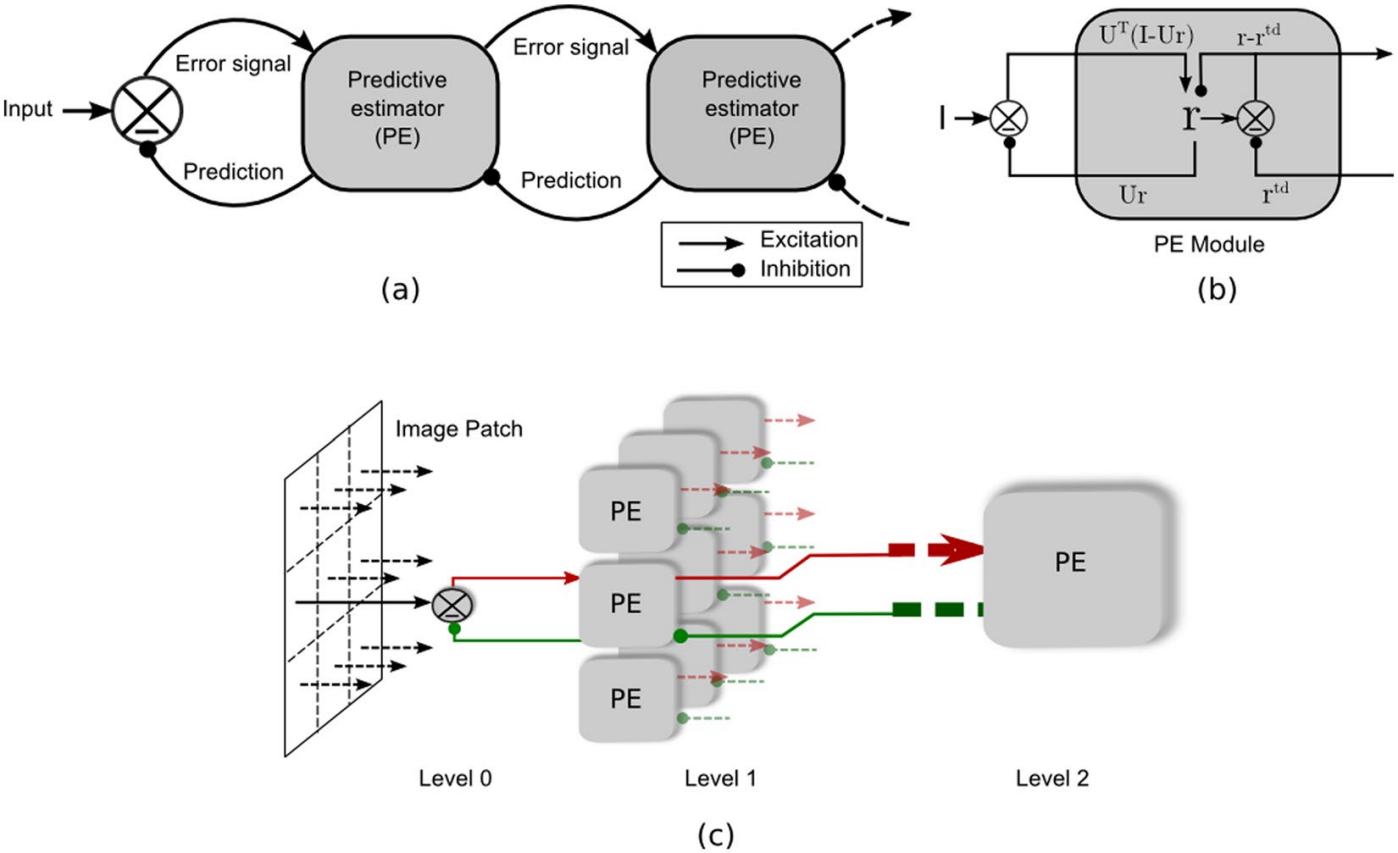
Hierarchical predictive coding (HPC)^20^. (**a**) The general mechanism of Hierarchical Predictive Coding is shown schematically. (**b**) The general computational architecture of a predictive estimator (PE) module is presented. (**c**) Details of a three level HPC model network is shown; where level 2 module sends a feedback signal to all 9 level 1 modules and in response, receives back the error signal from all of them.

In this paradigm, the visual system is considered to be an active predictor-corrector system implemented in a hierarchical neural architecture where perception is accomplished via the interaction of top-down prediction and bottom-up correction^21, 31^. Instead of passively responding to the input signal, higher-level cortical activities (predictions) are conveyed to lower levels via top-down connections, and in response, lower levels convey residual errors via bottom-up connections (see Fig. 9a). It is further assumed that prediction by the higher cortical levels is mainly governed by the regularities learned via the exposure to the natural scene during development.

The visual system learns the model of the outer world through its parameters related to statistical regularities **U**. The prediction **Ur** is generated from the activity of the neurons coding the internal representations or estimate **r** which is the actual cause of input sensory signal **I**. Given that the vision is a stochastic phenomenon, the goal of the visual system is, thus, to maximize the posterior probability distribution $P({\bf{r}},{\bf{U}}|{\bf{I}})$. According to the Bayesian theorem, this is roughly equal to the product of likelihood $P({\bf{I}}|{\bf{r}},{\bf{U}})$, which is a distribution of stochastic error between prediction and sensory input, and the prior distributions *P*(**r**) and *P*(**U**). Assuming Gaussian type stochastic error, with variance σ^2^, the posterior distribution can be written as -

1 $$P({\bf{r}},{\bf{U}}|{\bf{I}})=\frac{{\rm{1}}}{Z}\exp (-\frac{{|{\bf{I}}-{\bf{U}}{\bf{r}}|}^{{\rm{2}}}}{{\sigma }^{{\rm{2}}}})P({\bf{r}})P({\bf{U}})\,$$

where, *Z*, is a normalization constant. Maximizing this equation is equivalent to minimizing the negative logarithm of it, which is called cost function in the MDL terminology and can be written as,

2 $${E}_{{\rm{1}}}=\frac{{\rm{1}}}{{\sigma }^{{\rm{2}}}}{({\bf{I}}-{\bf{U}}{\bf{r}})}^{T}({\bf{I}}-{\bf{U}}{\bf{r}})+g({\bf{r}})+h({\bf{U}})$$

where the subscript *T* indicates the transpose of the vector or matrix. Also *g*(**r**), *h*(**U**) are the negative logarithm of *P*(**r**) and *P*(**U**), respectively.

The cost function of an inference system with 3 level of hierarchy, in which the higher (3rd) level makes inference (or prediction) **r** ^*td*^ to the immediate level representation **r** with error variance ${\sigma }_{td}^{\,\,2}$, can be written as (for details see refs 21, 31)

3 $${E}_{{\rm{2}}}=\frac{{\rm{1}}}{{\sigma }^{{\rm{2}}}}{({\bf{I}}-{\bf{U}}{\bf{r}})}^{T}({\bf{I}}-{\bf{U}}{\bf{r}})+\frac{{\rm{1}}}{{\sigma }_{td}^{\,\,2}}{({\bf{r}}-{{\bf{r}}}^{td})}^{T}({\bf{r}}-{{\bf{r}}}^{td})+g({\bf{r}})+h({\bf{U}})$$

This equation serves as a guiding principle for the standard Hierarchical Predictive Coding (see Fig. 9b), which assumes that the predictive estimator (PE) modules at each visual processing level send the prediction signal ${\bf{U}}{\bf{r}}$ to its immediate lower processing level via feedback connection. On the other hand, the lower levels send back the error signal $({\bf{I}}-{\bf{U}}{\bf{r}})$ via feed-forward connection. The error signal is then utilized to correct the current estimate **r**, which is coded by PE neurons, of the sensory driven input.

The dynamics and the learning rule, thus, result from minimizing the cost function (using gradient decent method), with respect to **r** and **U** respectively-

4 $$\frac{d{\bf{r}}}{dt}=-\frac{{k}_{{\rm{1}}}}{{\rm{2}}}\frac{\partial {E}_{{\rm{2}}}}{\partial {\bf{r}}}=\frac{{k}_{{\rm{1}}}}{{\sigma }^{{\rm{2}}}}{{\bf{U}}}^{T}({\bf{I}}-{\bf{U}}{\bf{r}})+\frac{{k}_{{\rm{1}}}}{{\sigma }_{td}^{\,\,2}}({{\bf{r}}}^{td}-{\bf{r}})-\frac{{k}_{{\rm{1}}}}{{\rm{2}}}g^{\prime} ({\bf{r}})$$

5 $$\frac{d{\bf{U}}}{dt}=-\frac{{k}_{{\rm{2}}}}{{\rm{2}}}\frac{\partial {E}_{{\rm{2}}}}{\partial {\bf{U}}}=\frac{{k}_{{\rm{2}}}}{{\sigma }^{{\rm{2}}}}({\bf{I}}-{\bf{U}}{\bf{r}}){{\bf{r}}}^{T}-\frac{{k}_{{\rm{2}}}}{{\rm{2}}}h^{\prime} ({\bf{U}})$$

Kurtosis prior probability $P({r}_{i})=\exp (-\alpha \,\mathrm{ln}({\rm{1}}+{r}_{i1}^{2}))$ on response *r*_i_ has been considered in this study to accommodate sparse coding^32^, which provides $g^{\prime} ({r}_{i})={\rm{2}}\alpha {r}_{i}/({\rm{1}}+{r}_{i}^{2})$. Additionally, considering prior $P({\bf{U}})$ as a Gaussian provides $h^{\prime} ({\bf{U}})={\rm{2}}\lambda {\bf{U}}$. Here $\alpha$ and $\lambda$ are variance related parameters.

An optimum estimate at a visual processing level is determined by the error signal from lower area (first term in the equation (4)) as well as error signal corresponding to a higher level (second term in equation (4)) that carry the contextual information since the higher area codes larger visual patch. This multilevel optimum-estimate for prediction is considered as an internal representation of the sensory input. The internal representation fabricated from the prediction ${\bf{U}}{\bf{r}}$ is assumed to represent ‘perceptual experience’ in this study.

### Network

A three-level network has been used in this study (Fig. 9c). Level 0, level 1 and level 2 are equivalent to the LGN, V1, and V2. Level 0 pre-procsses (low pass filtering) the stimuli in line with LGN function. Each module at level 1 sends prediction signal to level 0, by feedback connection and in response receives the error signal by the feed-forward connection. Likewise, each module at level 2 sends the prediction signal to all 9 modules at level 1, and get back the error signal by a feed-forward connection from all of them. The modules at level 1 consist of 130 feed-forward, 130 PE neurons, and 144 feedback neurons. The level 2 module contains 256 feed-forward neurons, 256 PE neurons, and 1170 feedback neurons.

#### Training

For obtaining statistically significant results, we performed 40 training cycles. In each training cycle, the network received a thousand batches of 100 (variance normalized and pre-processed^32^) 30 × 30 pixel image patches as inputs. Each level 1 module received signals corresponding to 12 × 12 pixel image patches which were overlapped by 3 pixels^20^. The network was allowed to achieve the optimum-estimate (equation (4)) for each batch, and then the average of the optimum-estimate was used to update the weighting profile of neurons (equation (5)), initially assigned to random values. To prevent the weighting profile from growing boundlessly, the gain of the weighting profile of each neuron were adapted such that it maintains the equal variances on the response. Parameters used in this study are same as considered in the previous study^20^.

### Blind spot implementation

First, the model network was trained without considering the blind spot, and thereafter, the blind spot was created in the trained network by removing the feed-forward connection from level 0 to level 1 (8 × 8 pixel wide in the middle of BS module). This process is in agreement with the actual physiological findings, where the neurons contributing to the filling-in process (at the blind spot) are found to be of a binocular type and therefore, receive inputs from both the eye. Thus, despite the absence of any input from one eye (the blind spot eye), the neurons could develop their weighting profiles. For a detailed discussion see the previous study^20^.
